# Cardiac myosin inhibitors: Efficacy, safety and future directions of aficamten in hypertrophic obstructive cardiomyopathy

**DOI:** 10.1186/s43044-025-00652-0

**Published:** 2025-06-13

**Authors:** Ikponmwosa Jude Ogieuhi, Victor Oluwatomiwa Ajekiigbe, Boluwaduro Abasiekem Adeyemi, Bright Nwatamole, Komolafe Babajide Ayodeji, Oshomoh Mark-Anthony Ugiomoh, Temiloluwa Adebayo Odeniyi, Adewunmi Akingbola, Efosa Peace Iyawe, Olabode Olawale Oladejo, Motunrayo Oluwatoyosi Lawal, Nathnael Abera Woldehana, Ifeoluwa Sandra Bakare, Adejumo Temilade Patience, Grace Chinenye Okoro

**Affiliations:** 1 Siberian State Medical University, Tomsk, Russian Federation; 2 Ladoke Akintola University of Technology, Ogbomoso, Nigeria; 3 Northwestern Medicine, McHenry, Chicago, USA; 4 Vassar Brothers Medical Center, Poughkeepsie, USA; 5 University of Lagos, Lagos, Nigeria; 6 Obafemi Awolowo University Teaching Hospitals Complex, Ile-Ife, Nigeria; 7 University of Cambridge, Cambridge, UK; 8 Babcock University Teaching Hospital, Ogun, Nigeria; 9 Johns Hopkins University, Baltimore, USA; 10 Ternopil State Medical University, Ternopil, Ukraine; 11 Ivano-Frankivsk National Medical University, Ivano-Frankivsk, Ukraine

**Keywords:** Aficamten, Hypertrophic obstructive cardiomyopathy, Clinical Trials, Efficacy, Cardiac Myosin Inhibitors

## Abstract

**Background:**

Hypertrophic obstructive cardiomyopathy (HOCM) is a genetic disorder that affects the cardiac myocytes leading to asymmetric hypertrophy of the left ventricle and obstruction of the left ventricular outflow tract (LVOT) with possible risk of sudden cardiac death in some high-risk patients. This paper aims to evaluate the efficacy, safety, and future prospects of aficamten in the management of HOCM and its potential to transform current standards of care.

**Main Body:**

Over the years, there have been significant milestones in HOCM management, including pharmacological and surgical techniques, which have reduced the morbidity and mortality associated with this disease. However, HOCM remains a challenging disease to manage for healthcare providers due to the heterogeneous nature of its presentation and limitations to the conventional treatment medications including beta blockers and non-dihydropyridine calcium channel blockers. These traditional options often provide inadequate symptomatic relief and have significant side effects. Surgical options such as septal ablation have shown positive outcomes but are associated with procedural risks. Therefore, recent advancements have led to the development of novel agents such as cardiac myosin inhibitors (CMIs).

**Conclusion:**

Aficamten is a new second-generation cardiac myosin inhibitor (CMI) that has shown promising results from clinical trials. With reports of reduced LVOT obstruction and improvement in heart failure symptoms, these findings indicate potential improvement in the quality of life of patients with HOCM.

**Graphical Abstract:**

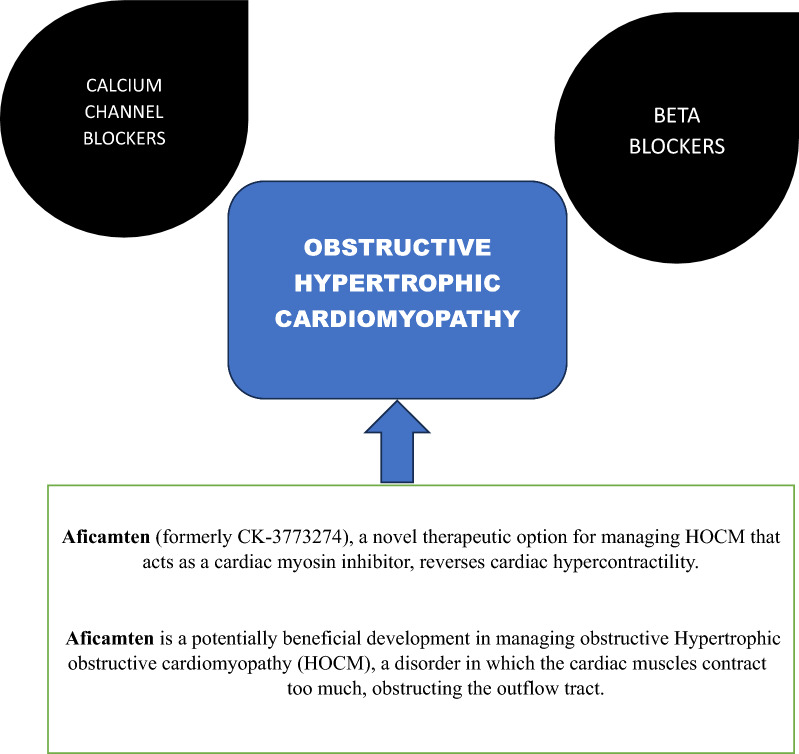

## Background

### Overview of hypertrophic obstructive cardiomyopathy

Hypertrophic obstructive cardiomyopathy (HOCM) is a subset of hypertrophic cardiomyopathy (HCM), an autosomal dominant cardiac disease characterized by mutations in sarcomere proteins leading to abnormal cardiac structural changes, with an estimated prevalence of about 1 in 500 adults [1]. According to 2020 American Heart Association/American College of Cardiology (AHA/ACC) guideline, HCM is a disease state distinguished largely by left ventricular (LV) hypertrophy “in the absence of another cardiac, systemic, or metabolic disease capable of initiating the extent of hypertrophy seen in such patient and for which a disease-causing sarcomere (or sarcomere-related) variant is identified, or genetic etiology remains unresolved” [2]. HCM may be obstructive or nonobstructive and about seventy percent of patients with HCM have the obstructive type, where there is asymmetric LV hypertrophy leading to obstruction of the left ventricular outflow tract (LVOT). In contrast, the remaining patients with HCM have the non-obstructive type with no LVOT obstruction [1]. A considerable number of cases of sudden cardiac death in young people, especially young athletes, have been discovered to be due to HOCM. However, it remains asymptomatic and undiagnosed in some persons until an adverse cardiac event occurs [3, 4].

The pathophysiology of HOCM is based on asymmetric hypertrophy of the LV leading to LVOT obstruction, consequently reducing stroke volume and cardiac output [5]. One of the mechanisms that potentiates LVOT obstruction in HOCM is the excessive actin-myosin cross-bridges within the cardiomyocyte sarcomeres leading to cardiac hypercontractility [6]. It may be associated with syncope, heart failure [7], and atrial fibrillation, which increases the risk of cardioembolic stroke [8].

### Current challenges in HOCM management

Over the years, significant milestones have been attained in the management of HOCM, leading to reduced morbidity and mortality from the disease. HOCM diagnosis is a challenge because the symptoms of this disease may mimic symptoms of many other cardiovascular disorders so the diagnosis may be delayed. For the diagnosis of HOCM in adults, the European Society of Cardiology recommends using a left ventricular wall thickness of ≥ 15 mm on echocardiography with a nondilated, hyperdynamic left ventricle. However, this is not always found on echocardiography of patients with HOCM, with a diagnostic sensitivity of about 80% [9].

The conventional pharmacological therapies for symptomatic patients with HOCM include beta‐blockers, non‐dihydropyridine calcium channel blockers (CCBs), and disopyramide [10]. However, these drugs come with the challenge of adverse drug effects, inadequate supplies, and reduced symptom control over time [11, 12]. Although surgical management such as septal reduction therapy (SRT), surgical myectomy [13] or alcohol septal ablation [14] have shown positive outcomes potentially changing the natural progression of the disease, these procedures are associated with serious morbidity [15].

### Cardiac myosin inhibitors: a novel therapeutic option for HOCM

Direct inhibition of cardiac sarcomere contractility is a promising method of managing patients with HOCM by re-establishing the appropriate degree of contractility within the cardiac sarcomere [16, 17]. Recent advancements have led to the development of novel agents such as cardiac myosin inhibitors (CMIs). Aficamten is a new second-generation CMI that has shown promising results from clinical trials. It is a novel therapeutic option for managing HOCM that works mainly by reversing cardiac hypercontractility [18]. Aficamten reduces the activity of ATPase by slowing down the release of phosphate and making the actin-binding site more stable. Consequently, aficamten reduces the number of functional myosin heads, shortening the sarcomere [19]. Thus, it relieves left ventricular obstruction and improves the diastolic function of HOCM patients [20]. The phase 2 trial REDWOOD-HCM Cohorts 1–3 and the phase 3 trial SEQUOIA-HCM studies showed that aficamten offers both safety and pharmacodynamic effectiveness in patients with even the most intractable degrees of HOCM [18, 21].

### Mechanism of action of Aficamten

**Fig. 1:**
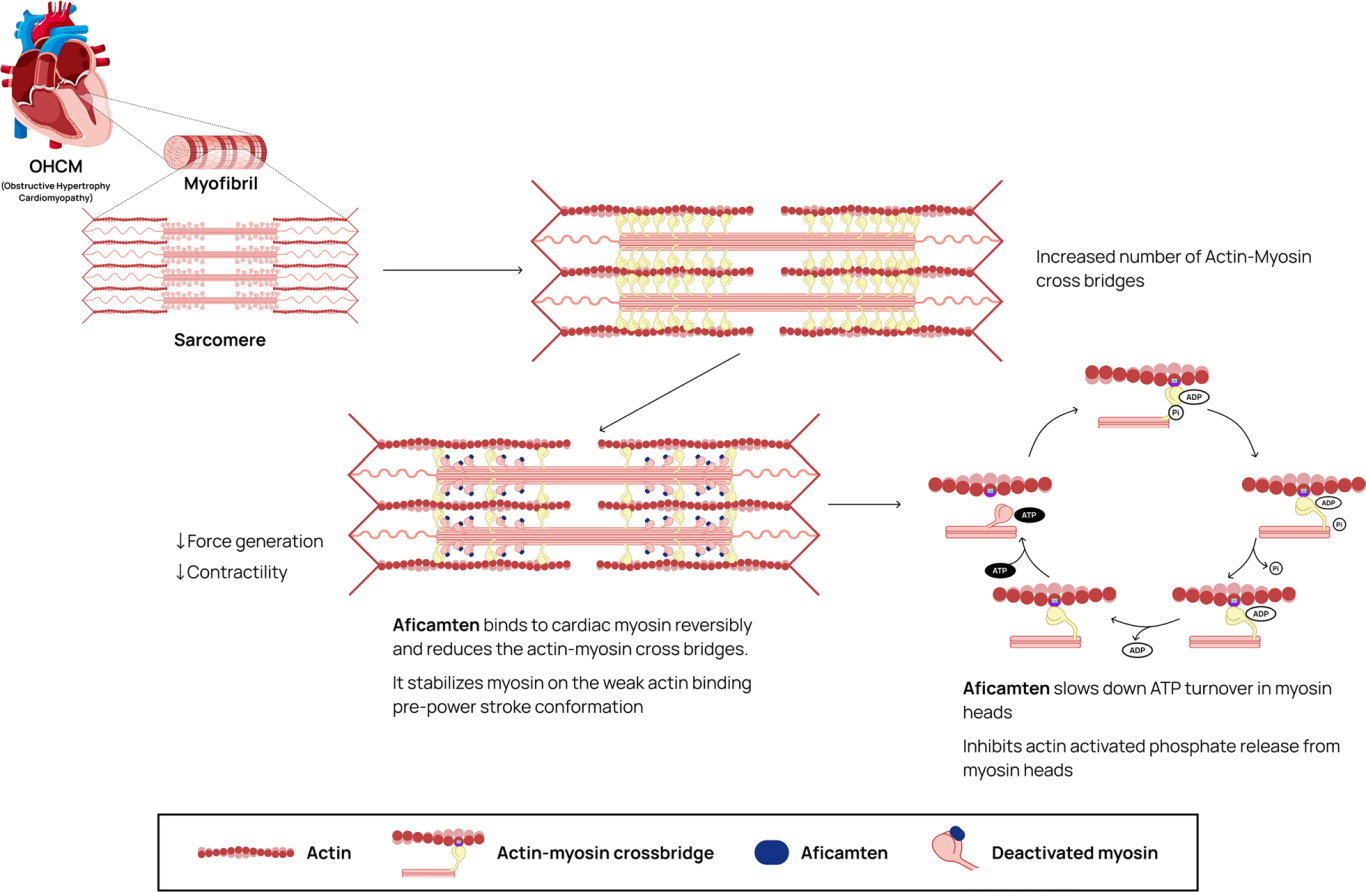
Mechanism of action of aficamten

Aficamten modifies cardiac contractility by interacting with cardiac myosin at a specific allosteric binding site [22]. (See Fig. 1) By selectively binding to cardiac myosin, aficamten inhibits the activity of cardiac myosin ATPase. This ATPase activity is essential for the hydrolysis of ATP in order to drive the conformational changes in myosin heads that are required for contraction [23]. Aficamten initiates the inhibition of this activity, lowering the energy available for the power stroke, and decreasing the force of contraction [23]. Aficamten's suppression of ATPase activity has an additional effect on the general dynamics of the heart's contractile apparatus. Since ATP is necessary for the myosin heads to separate from actin filaments after the power stroke, a reduction in ATPase activity lengthens the time in which this contact occurs. As a result of this extended engagement, there is less cross-bridge cycling since there is a lower frequency of cross-bridge detachment [23]. The hallmark of HOCM is the genetic abnormality impacting contractile protein causing aberrant increase in myocardial contractility [5]. By directly targeting the hyperactive myosin, aficamten counteracts this hypercontractile condition and restores contractility to normal, resulting in reduced obstruction of the LVOT, an improved diastolic function, and a reduction in the level of oxygen demand by the heart [24–26].

Since Aficamten shows great prospects in the management of HOCM, this review aims to evaluate the efficacy, safety, and future prospects of aficamten in the management of HOCM and its potential to transform current standards of care.

## Main text

### Methodology

**Fig. 2:**
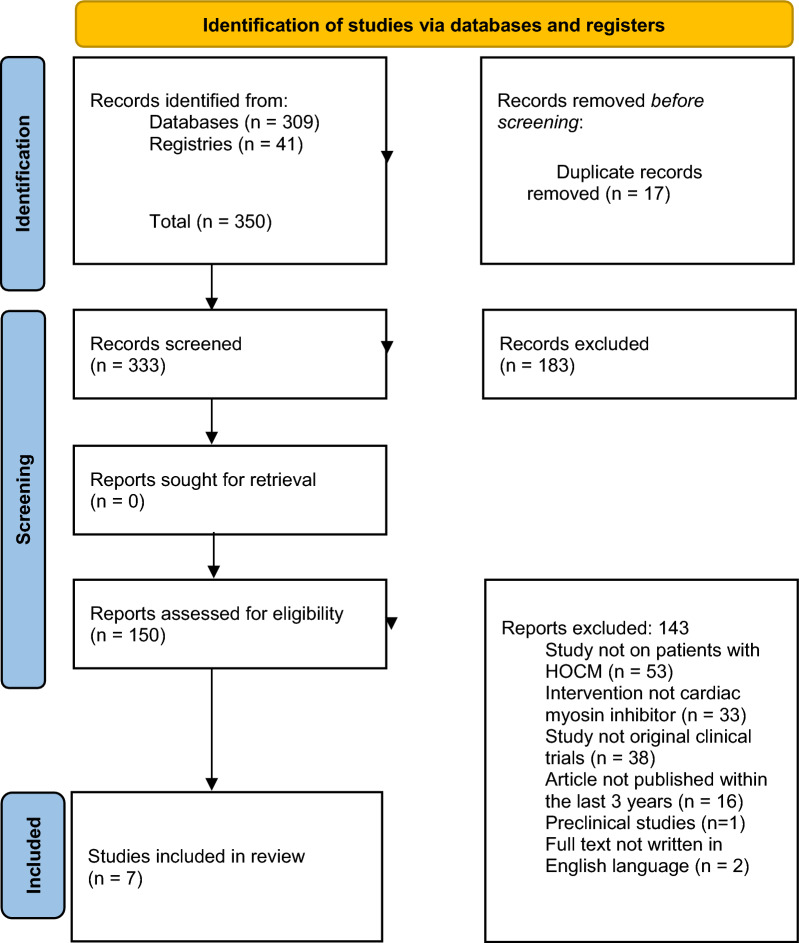
PRISMA flowchart summarizing the selection process

A comprehensive search was conducted using PubMed, Google Scholar, Cochrane Library, Directory of Open Access Journals, and ScienceDirect. Our search strategy was guided by key concepts, MeSH terms, and free terms such as ‘Aficamten,’ ‘Obstructive Hypertrophic Cardiomyopathy,’ and ‘Clinical Trials.’ We also combined them with appropriate Boolean operators (AND, OR, NOT) (Fig. 2).

Table 1 contains seven (7) randomized control trials published between 2022 and 2025 that summarize the efficacy and safety profile of aficamten.

**Table 1:** Overview of Clinical Trials published between 2022 and 2024 that summarize the efficacy and safety profile of Aficamten

S/N	Author/year	Study design	Sample size	Study duration	Meanage	Sex	Efficacy	Safety and adverse effects	Other outcomes
1	Zhao et al., 2023 [20]	Double-blinded, randomized, placebo-controlled, Phase 1 study	28 patients	1 month	31.6 years	22 males	Aficamten demonstrated dose-proportional effects with doses ranging from 5–20 mg and with accumulated effects noticed with multiple dosing. Increasing plasma concentrations of Aficamten led to reduction in LVEF and LVFS, consistent with its intended pharmacological effect	Aficamten was safe and well-tolerated in healthy Chinese participants, with no serious adverse events or treatment discontinuations due to adverse events. The most common treatment-related side effects were mild, including systolic hypotension and mild conjugated hyperbilirubinemia. These effects were transient and resolved without sequelae	While hyperbilirubinaemia was more frequent in Aficamten groups, it was mild and bilirubin levels rapidly returned to baseline
						6 females			
2	Maron et al., 2023 [21]	Phase II, multicenter, randomized, placebo-controlled, double-blind, dose-finding study	69 Patients	15 months and 25 days	57 years	31 males	Aficamten significantly reduced LVOT gradients in patients with HOCM, with most patients achieving complete hemodynamic response (resting LVOT gradient < 30 mmHg and Valsalva LVOT gradient < 50 mmHg). This was accompanied by clinical improvements including reduced heart failure symptoms and reductions in cardiac biomarkers like NT-proBNP.	Aficamten was tolerated with ease. There were no treatment-related serious adverse events, no adverse events leading to early treatment termination or interruption. The majority of adverse events were described as moderate or mild	All patients except one placebo recipient completed the study, with nearly all data points available for analysis. Three patients experienced side effects including stress cardiomyopathy and exacerbation of pre-existing back pain but none were considered related to the study drug by investigators
						38 females			
3	Maron et al., 2024 [22]	Phase 3, international, double-blind, randomized, placebo-controlled trial	282 patients	15 months, 14 days	59.2 ± 12.6 years	167 males	Aficamten significantly improved peak oxygen uptake by 1.7 ml/kg/min compared to placebo at 24 weeks (p < 0.001) in patients with symptomatic obstructive hypertrophic cardiomyopathy. The drug also met all 10 secondary endpoints, including reductions in LVOT gradients and improvements in symptoms and quality of life	Aficamten demonstrated a favorable safety profile, with adverse event rates similar to placebo (73.9% vs 70.7%). Only one patient discontinued Aficamten due to an adverse event (paranoia). A transient reduction in LVEF below 50% occurred in 3.5% of Aficamten patients, but none required treatment interruption or experienced heart failure exacerbation	A transient reduction in LVEF to less than 50% occurred in 3.5% of the Aficamten group versus 0.7% of the placebo group, but this did not lead to treatment interruptions or heart failure exacerbations
						115 females			
4	Malik et al., 2022 [25]	Randomized, placebo-controlled, single ascending dose (SAD) and multiple ascending dose (MAD) design	102 patients	13 months and 26 days	32–40 years	77 males	The study found that Aficamten was well-tolerated and demonstrated dose-dependent reductions in LVEF at pharmacologically active doses. Single doses up to 50 mg and daily doses up to 10 mg for 14–17 days appeared safe	Aficamten was generally well-tolerated, with adverse events mostly mild and comparable to placebo in frequency. The main safety finding was dose-dependent, reversible reductions in LVEF, with a few participants experiencing decreases up to < 50% that resolved within hours	The secondary goal of the trial, which was to determine a pharmacologically active dose and describe its pharmacokinetic/pharmacodynamic relationship, was accomplished
						25females			
5	Masri et al. [27]	open-label phase 2 trial	40 patients	8 months, 1 day	55.9 years	16 males	The study found that Aficamten treatment was associated with improvements in heart failure symptoms and reductions in cardiac biomarkers in patients with nonobstructive hypertrophic cardiomyopathy. After ten weeks of treatment,, 55% of patients experienced an improvement of ≥ 1 NYHA class, and there were significant reductions in NT-proBNP (56%) and high-sensitivity cardiac troponin I (22%) levels	Aficamten was generally well tolerated, with most adverse events reported as mild or moderate. had a fatal arrhythmia during the study	Three patients experienced asymptomatic reduction in left ventricular ejection fraction < 50%, which returned to normal after washout, and one patient with a history of aborted sudden cardiac death had a fatal arrhythmia during the study
						24 females			
6	Owens et al., 2023 [28]	RCT	13 patients	3 months	62 years	6 males	Aficamten demonstrated efficacy in reducing LVOT gradients and improving symptoms in patients with refractory obstructive hypertrophic cardiomyopathy already taking disopyramide. The drug appeared to be safe, with no serious adverse events when co-administered with disopyramide	Aficamten was well-tolerated with no serious adverse events, dosage interruptions, or treatment discontinuations reported. There were no meaningful changes in QTc intervals or vital signs, and no patients experienced left ventricular ejection fraction below 50%	The modest reduction in LVEF was reversible, returning to baseline after a 2-week washout period. The safety profile was consistent with findings from previous cohorts in the REDWOOD-HCM study. Overall, coadministration of Aficamten with disopyramide appeared to be well-tolerated in this patient population
						7 females			
7	Coats et al., 2024 [18]	Multicenter, randomized, double‐blind, placebo‐controlled, phase 3 trial	280 patients	15 months, 14 days	58.1 ± 12.7 years	165 males	The study found that Aficamten was effective in reducing LVOT gradient in patients with HOCM, with a favorable safety profile. A site-based dosing algorithm targeting the lowest effective dose of Aficamten successfully reduced obstruction while maintaining LVEF ≥ 50% in most patients	Treatment-emergent adverse events were similar between Aficamten and placebo groups, and there were no treatment interruptions or heart failure worsening for LVEF < 50%	Aficamten demonstrated a favorable safety profile with no major adverse cardiovascular events associated with treatment
						115 females			

*HOCM* Hypertrophic Obstructive Cardiomyopathy, *LVEF* Left Ventricular Ejection Fraction, *LVOT* Left Ventricular Outflow Tract, *NT-proBNP* N-terminal pro brain natriuretic peptide, *NYHA* New York Heart Association

## Inclusion criteria:

- Human clinical trials, randomized controlled trials and observational studies that focused on aficamten.
- Studies published in English Language
- Studies focused on the use of aficamten for HOCM management

## Exclusion criteria:

- Opinions, comments, reviews, case reports, systematic reviews or meta-analyses
- Studies not containing participants diagnosed with HOCM
- Studies conducted on animals
- Studies that used other related novel treatments
- Studies not published in English Language

Data from the databases were saved as comma-separated value files (CSV) and imported into the Rayyan software tool to remove duplicates. Further screening was done by two reviewers, V.O.A and O.U.M, and concerns were resolved by a third reviewer, I.J.O. Data was subsequently extracted onto an Excel spreadsheet to include author/year, study design, sample size, study duration, objective, sex, efficacy, and safety profile (Table 1). Finally, by utilizing narrative synthesis, we critically evaluated the current evidence of derived data.

## Results

We reviewed seven clinical trials regarding efficacy and safety profiles of aficamten published between 2022 and 2024. Out of these, two were phase 3 randomized, double-blinded, placebo-controlled trials running for about 15.5 months with about 280 patients each [18, 22]. We reviewed one phase 2 multicenter, randomized, double-blinded, placebo-controlled, dose-finding trial which was done across nearly 16 months with 69 patients and one open-label phase 2 trial with 40 patients studied across eight months [21, 27]. The other studies consisted of two phase 1 trials [20, 25] and one open-label trial with 13 patients across three months [28]. All seven studies demonstrated significant benefits with aficamten use (Table 1).

Maron et al., 2024 showed that among 282 patients (167 of whom were male), aficamten improved peak oxygen uptake by 1.8 ml/kg/min after 24 weeks compared to no change with placebo (1.8 ml/kg/min, 95% CI 1.2 – 2.3 in the aficamten group versus 0.0 ml/kg/min, 95% CI − 0.5 to 0.5 in the placebo group) [22]. This was accompanied by statistically significant improvements in quality of life, NYHA functional class, and LVOT gradient (LVOT-G) [22]. Similarly, Coats et al., 2024 in their study with 280 patients (165 of whom were male), showed a statistically significant reduction in LVOT-G, heart failure symptoms, and health status, with LVOT-G improving rapidly within two weeks. Kansas City Cardiomyopathy Questionnaire-Clinical Summary Score (KCCQ-CSS) improved by 7 points in the aficamten group compared to the placebo group (95% CI, score difference = 5–10). Furthermore, 58% of patients receiving aficamten versus 24% of patients receiving placebo had ≥ 1 NYHA class improvement, and Valsalva LVOT-G decreased by 50 mmHg (95% CI, 44–57 mmHg) in the aficamten group compared to placebo group [18].

As summarized in Table 1, trials by Maron et al. and Masri et al. showed significant improvements in NYHA classes and health status measured by the KCCQ-CSS [22, 27]. Both trials conducted by Zhao et al. and Malik et al. showed reductions in LVEF correlating with the dosage of aficamten, consistent with its mechanism as a CMI [20, 25]. In their open-label trial, Owens et al., 2023 showed that aficamten produced significant reductions in LVOT-G, modest and reversible LVEF reductions, and improvements in NYHA functional classes [28].

Aficamten offered the aforementioned benefits while maintaining a good safety profile, with side effects that were mostly mild to moderate, similar to those found in placebo groups. None of the seven trials were terminated due to adverse effects, with very few serious adverse events reported. There were four reports of serious adverse events in one study but they were deemed unrelated to aficamten [27]. The maintenance of LVEF was often reported as a significant safety parameter. For instance, two trials conducted by Coats et al. and Maron et al. reported mild to modest, transient, reversible, dose-dependent reductions in LVEF which did not lead to treatment interruptions or decompensated heart failure [18, 22]. This was observed in 3.5% of patients in one trial (with a reduction in LVEF to < 50%) [20] and 4.9% of patients in another trial who underwent per-protocol dose reductions due to LVEF < 50% [18].

Malik et al., 2022 reported that among patients with LVEF < 50%, there were no adverse changes in vital signs or symptoms, and the LVEF in these individuals reverted to baseline within a day [25]. Maron et al., 2024 reported that palpitations (7.0% vs. 2.9%) and hypertension (7.7% vs. 2.1%) were more common in patients receiving aficamten than placebo [22], and this was assumed to be likely related to potential rebound during washout phase. However, these symptoms were not severe enough to necessitate the withdrawal of the drug [18]. Notably, Zhao et al., 2023 reported decreased rather than increased systolic blood pressure among patients using aficamten, although the blood pressure returned to baseline after discontinuation [20]. The study also reported slight increases in bilirubin that were more common in the aficamten group but reversible after discontinuation of the medication. Of the seven trials reviewed, four studies reported that aficamten was well tolerated overall [20, 21, 25, 27].

## Discussion

In the past, limited understanding of the disease has impeded the development of disease-modifying therapies for HOCM. The exploration of the CMIs such as mavacamten and aficamten aimed to improve the symptoms and modify the disease process following insight from the genetic and pathophysiology of HOCM. Recommended guidelines for pharmacological therapy of symptomatic HOCM include pharmacotherapies such as beta-blockers, calcium channel blockers, and disopyramide [29]. These drugs typically do not offer complete relief of symptoms, and there is little to no evidence that they prevent disease progression or alter the natural course of the disease. These drugs also have side effects that may impair the quality of life [29]. For instance, disopyramide is associated with prolonged QTc and disturbing effects due to its anticholinergic properties [30]. Most of these side effects have not been reported in patients on the therapeutic doses of aficamten. The 2023 European guidelines recommend CMIs as a second-line therapy when beta-blockers, calcium channel blockers, and/or disopyramide are poorly tolerated or ineffective. As the understanding of aficamten keeps evolving, there is continual incorporation of the medication into HOCM guidelines. The ongoing phase III trial, MAPLE-HCM, continues to assess the efficacy of aficamten as compared to Metoprolol as monotherapy for patients with HOCM [31].

### Role of Aficamten in symptom relief and functional improvement, and potential impact of Aficamten on HOCM management

The results of the aficamten clinical trials have been promising, with evidential insights into the subjective and objective improvements reported among patients with HOCM**.** In the REDWOOD – HCM phase II clinical trial, aficamten significantly improved in all relevant secondary and exploratory endpoints compared with placebo [27]. After two weeks of starting the medication, there was a slight decrease in LVOT-G, with most patients experiencing an LVOT-G of < 30mmHg. Aficamten was noted to reduce the gradient below the guideline-directed threshold for invasive SRT [27]. Aficamten also improved the symptoms of heart failure, with over 50% of patients experiencing a change of ≥ 1 NYHA functional class [22]. The findings from aficamten trials are encouraging and would potentially improve the quality of life of patients with HOCM who continue to experience limitations from their symptoms despite conventional therapies. However, more clinical studies are needed before incorporating this medication into the current guidelines for HOCM treatment. Improved understanding of the genetic basis and pathophysiology of HOCM has paved the way for the development of novel medications. The traditional therapies in the management of HOCM focus on symptom control, quality of life improvement, and reduction of the risk of sudden cardiac death but without disease-modifying capacity [29]. Despite the combination of these medications, at least a third of patients with HOCM end up receiving surgical intervention and SRT due to the refractoriness of their symptoms [32]. While SRT may have excellent operative outcomes, it does not modify the disease's underlying pathophysiology [33]. Furthermore, SRT is associated with significant mortality and morbidity, with a recorded 1% to 2% mortality rate even when performed by the best experts [34]. The discovery of aficamten has opened a new era in caring for patients with HOCM. Compared with mavacamten, another novel CMI, aficamten has shown a good safety profile, with no evidence of P450 enzyme induction and drug interactions, a long washout period, and a longer half-life. Hence it opens more options for clinicians and patients when discussing treatment strategies for HOCM [29]. Some studies have ascertained risk of sudden cardiac death in HOCM using the peak oxygen uptake (pVO2). The measurement of the pVO2 during cardiopulmonary exercise testing (CPET) is the gold standard for determining oxygen uptake during maximal exercise, which directly correlates with cardiac output [33]. The pVO2, in addition to measuring the functional capacity more effectively than subjective measures of the NYHA functional class, has frequently been shown to predict clinically relevant outcomes in both HOCM and non-obstructive HCM. A study has shown that the risk of death or procedural intervention in HOCM was reduced by 21% for each 1ml/kg/min increase in pVO2 [36]. Both SRT and CMIs have been shown to improve PVO2, unlike beta-blockers [33]. These findings highlight an additional objective marker that supports the functional and prognostic benefit of aficamten in HOCM management.

### Potential to reduce the need for surgical interventions

In the management of HOCM, when the medical therapy has failed to control the symptoms despite maximal drug optimization SRT is considered. SRT can be done by either surgical myectomy or alcohol septal ablation. While the surgical mortality rate may be less than 1% in experienced high-volume centers, most SRT done in lower-volume centers has perioperative deaths as high as 15% [34]. Although SRT is currently the second-line therapy for lower-risk patients, the unavailability of experienced centers for SRT may provide the avenue for using CMIs as an initial strategy, irrespective of the procedural risk, until access to the experienced center is established [34]. Other considerations like recovery time and hospital stay may pose a challenging logistic challenge to young patients who are actively working or have limited time due to schedules [34]. Considering these, the CMI may serve as a temporary therapy until SRT becomes more accessible or convenient. CMIs may also be considered for those with higher operative risk due to unsuitable anatomy [35]. Furthermore, studies have shown that aficamten can reduce the left-ventricular outflow gradient to levels below the threshold for SRT in some patients. While surgery remains an essential treatment option, especially in those with concomitant structural cardiac abnormalities such as atrial fibrillation or valvular disease, CMIs like aficamten may help reduce the immediate need for surgical intervention in selected patients.

### Limitations faced among trials on Aficamten in HOCM

In a study encompassing 101 sites in 14 countries, 543 patients were screened for eligibility, of which 282 went through randomization and were given either placebo or aficamten. This reduction in participants was based on the selection criteria, of which participants who had an inadequate elevated LVOT gradient following the Valsalva maneuver or who failed the cardiopulmonary exercise testing criteria were screened out. Also, of the 282 patients, only 273 completed the trial because nine patients discontinued the treatment [22]. There was also a limitation in the racial diversity, as not all races were represented in the studies. With HOCM being ubiquitous among various ethnic groups [38–44], this may be a barrier to the generalization of use of aficamten as there could be genetic variations in its pharmacodynamics.

### Safety profiles of Aficamten

Specific adverse events were noted among patients in both the placebo and aficamten groups. Some of these side effects include atrial fibrillation, ventricular fibrillation, palpitation, and hypertension and most of them appeared more among patients in the aficamten group but resolved upon discontinuation of the drug [22]. Importantly, compared to mavacamten, with a half-life of 6–9 days, aficamten exhibits a short half-life of approximately two days, allowing a faster excretion rate in the face of side effects [45]. Furthermore, this property enables it to reach effective doses within a few weeks, thereby contributing to certain clinical benefits. Compared to mavacamten, aficamten was also found to not exhibit interactions with certain liver enzymes (CYP2C19 and CYP3A4), which reduces the chances of drug-drug interaction contributing to its safer and more efficient use in treating HOCM [46].

### Future directions

Aficamten may have potentially positive effects among patients even beyond addressing HOCM symptoms; however, this needs to be studied more. For instance, the REDWOOD-HCM trial, specifically Cohort 4, which revealed encouraging results in reducing heart failure symptoms, echocardiographic markers, and cardiac biomarkers in non-obstructive HCM was done over a short period [27]. This short study duration highlights the need for longer-term investigations in order to understand the long-term benefits of aficamten in this population.

There is potential for combining aficamten with standard therapy. As shown in the SEQUOIA-HCM trial, aficamten has demonstrated promising results in improving exercise tolerance regardless of beta-blocker use. This contrasts with mavacamten, which may be less effective when used with beta-blockers, as revealed in the EXPLORER-HCM trial [16]. The potential to use aficamten as either monotherapy or in combination could enhance its ease of use in clinical practice as a standard treatment for HOCM. This represents a new area for researchers to look onto in future studies.

The development of aficamten and Mavacamten emphasizes the importance of precision medicine in advancing medical therapies. Precision medicine can facilitate the creation of targeted therapies for specific diseases, thereby improving patient outcomes and addressing unmet medical needs. In the context of HOCM, CMIs can target sarcomeric protein mutations that contribute to the disease, allowing for more personalized treatment approaches [47]. However, future research should focus on understanding which genetic variants respond favorably to aficamten and which do not, thereby refining treatment strategies and enhancing their effectiveness.

The FOREST-HCM trial demonstrated that 42% of patients eligible for SRT became ineligible after receiving aficamten for 36 weeks [48, 49]. This suggests that aficamten could be a valuable treatment option to reduce the need for invasive procedures. Although Mavacamten also showed promise in reducing SRT requirements in the VALOR-HCM trial, it is considerably more expensive than SRT [50]. Hence, it is essential to improve cost-effectiveness when pricing aficamten and future therapies.

CMIs could become the new standard of care. While Mavacamten has already received FDA approval, aficamten is currently under review and has been granted breakthrough therapy designation from the FDA and National Medical Products Administration (NMPA) due to promising clinical trial results [37]. If approved, aficamten could offer a new, less invasive treatment option for patients with HOCM. This could lead to a paradigm shift in clinical guidelines and a more personalized approach to managing the condition.

## Conclusions

Clinical trials have shown that the most efficient result of aficamten is a significant reduction in LVOT gradient, reducing stress to the heart. It addresses the cause of hypercontractility in the heart muscle and lowers LVOT gradients, attenuating cardiac hypercontractility. As a result, it improves exercise tolerance by reducing symptoms associated with exercise, including fatigue, chest pain, and dyspnea on exertion. Importantly, aficamten has shown a favorable safety profile with minimal adverse effects, making it a promising alternative to more invasive therapies like SRT. The future of HOCM treatment may eventually depend on aficamten as it is still being researched. Current observations examine its sustainability over time and possible synergies with other existing treatment methods. Aficamten may become part of the new standard of care for patients subject to ongoing research, decreasing the potential need for surgical interventions. The drug shows great potential to change the paradigm of care for those with HOCM and benefit their quality of life. Future studies should focus on broader patient populations targeting more races and ethnic groups in order to enable a more generalizable use of the medication.

## Data Availability

No datasets were generated or analysed during the current study.
